# TLR9 Signaling Protects Alcohol-Induced Hepatic Oxidative Stress but Worsens Liver Inflammation in Mice

**DOI:** 10.3389/fphar.2021.709002

**Published:** 2021-06-28

**Authors:** Liuyi Hao, Wei Zhong, Xinguo Sun, Zhanxiang Zhou

**Affiliations:** ^1^ Center for Translational Biomedical Research, The University of North Carolina at Greensboro, Kannapolis, NC, United States; ^2^ Department of Nutrition, The University of North Carolina at Greensboro, Kannapolis, NC, United States

**Keywords:** TLR9, oxidative stress, hepatocyte injury, mitochondrial dysfunction, ARLD

## Abstract

Toll-Like Receptor 9 (TLR9) elicits cellular response to nucleic acids derived from pathogens or dead cells. Previous studies have shown that TLR9-driven response may lead to differential impact on the pathogenesis of liver diseases. This study aimed to determine how TLR9 may contribute to chronic alcohol exposure-induced liver pathogenesis. We observed that *TLR9* KO mice were more susceptible to alcohol-induced liver injury, which was evidenced by higher serum ALT/AST levels and more lipid accumulation in alcohol-fed *TLR9* KO mice than wild-type mice. Alcohol-induced oxidative stress and mitochondrial dysfunction were also exacerbated by *TLR9* KO. We found that chronic alcohol exposure-induced hepatic CHOP and ATF6 activation were enhanced in *TLR9* KO mice. By using primary hepatocytes and AML-12 cells, we confirmed that TLR9 activation by CpG ODN administration significantly ameliorated acetaldehyde-induced cell injury via suppressing ATF6-CHOP signaling. By using *STAT3* knockdown AML12 cells, we showed that TLR9-mediated STAT3 activation inhibited ATF6-CHOP signaling cascade and thereby protecting against acetaldehyde-induced mitochondrial dysfunction and cell injury. Interestingly, we found that *TLR9* KO mice ameliorate chronic alcohol exposure-induced *CXCL1* induction and neutrophils infiltration in the liver. Furthermore, hepatocyte lack of *STAT3* significantly ameliorated CpG ODN and LPS-increased *CXCL1* levels in hepatocytes. Overall, our data demonstrate that TLR9 signaling in hepatocytes counteracts alcohol-induced hepatotoxicity but worsens proinflammatory response.

## Introduction

Chronic alcohol consumption leads to alcohol-related liver disease (ALD), which accounts for nearly half of liver cirrhosis-associated mortality in the United States (Gao and Bataller, 2011). Alcohol-induced simple fatty liver (steatosis), an early stage of ALD, is reversible; however, continued excessive alcohol intake can lead to progressive hepatitis and liver fibrosis. In some cases, the disease further progresses to cirrhosis and even hepatocellular carcinoma (Osna et al., 2017). Despite the disease progression was well known and the understanding of its pathogenesis was improved, the complex interlinked molecular events and related cellular behaviors that occurring during the pathogenesis of ALD have not been fully elucidated, hindering the development of effective clinical treatment for this disease.

Ethanol is metabolized mainly in the liver through alcohol dehydrogenase (ADH), cytochrome P450 2E1 (CYP2E1), and catalase pathways, which all generate toxic acetaldehyde (Setshedi et al., 2010). Acetaldehyde is a highly reactive aldehyde that can promote adduct formation, leading to functional impairments of key proteins, including enzymes, as well as DNA damage, which promotes oxidative stress, mitochondrial dysfunction, and ER stress in hepatocyte (Hao et al., 2018). Our previous studies demonstrated that accelerates acetaldehyde metabolism in the liver strongly ameliorates alcohol-induced liver injury in mice (Zhong et al., 2015).

Hepatocyte is densely packed with mitochondria, which is the principal site of adenosine triphosphate generation, and integrates cell metabolism by regulating both anabolic and catabolic pathways (Lee and Sokol, 2007). Alcohol-induced mitochondrial dysfunction appears to play a major role in the mechanisms by which alcohol causes liver injury (Mansouri et al., 2018). Furthermore, acetaldehyde induces ROS overproduction and impairs mitochondrial function, causing apoptosis and cytotoxicity of hepatocyte cells (Sun et al., 2016). Moreover, alcohol or acetaldehyde-induced mitochondrial dysfunction can augment oxidative stress, which, in turn, further promotes hepatocyte apoptosis in the liver, forming a vicious cycle (Tan et al., 2020). Early studies, including ours, revealed that mtROS scavenger (MitoQ) administration strongly ameliorated alcohol-induced hepatic steatohepatitis, suggesting alcohol-induced mtROS overgeneration and related oxidative stress plays a detrimental role in the development of ALD (Hao et al., 2018).

Inflammatory processes due to microbial dysbiosis, loss of barrier integrity in the intestine, hepatocellular stress and death, as well as inter-organ crosstalk have been recognized as the primary contributors to the development and progression of ALD (Lu et al., 2018; Zhong et al., 2019). However, recent studies indicated that the cytokines and small molecules that activate the inflammatory pathway may have a therapeutic potential to treat fatty liver disease (Gao, 2004; Wang et al., 2011). Furthermore, the host’s innate immune response is necessary to eradicate invading pathogens, which are harmful and will lead to oxidative stress-induced tissue injury, in part, by damaging mitochondrial structure and function (Mogensen, 2009; Hansen et al., 2015). Recognition of microbial pathogens is mediated by germline-encoded pattern recognition receptors, including toll-like receptors (TLRs). TLR9, which shows affinity toward both pathogen-derived and endogenous host DNA, is considered to play a crucial role in both DMAPs/PAMPs-induced liver injury by causing neutrophils activation and inflammatory cytokine/chemokine release in ALD (Roh et al., 2015). However, a previous study demonstrated that TLR9 signaling activation protects tert-Butyl hydroperoxide-mediated oxidative stress and cell death in macrophages (Qu et al., 2020). Furthermore, the whole body lack of TLR9 exacerbated diet-induced insulin resistance and obesity in mice (Hong et al., 2015). These findings suggest that TLR9 signaling-mediated cellular functions may largely depend on the type of cell and stress. Whether TLR9 regulates alcohol-induced hepatic oxidative stress and was still unclear. In this study, we aimed to investigate whether and how TLR9 can regulate hepatic redox balance and mitochondria functions in the pathogenesis of ALD.

## Materials and Methods

### Animal Study and Alcohol Feeding

NIAAA model was employed as previously described (Bertola et al., 2013) with some modifications. Twelve weeks old *TLR9* KO and C57BL/6 male mice were fed with the Lieber-DeCarli liquid diets containing alcohol (alcohol-fed; AF) or isocaloric maltose dextrin control liquid diet (pair-fed; PF) for 8 weeks. 4 h before tissue collection, the AF mice were gavaged with one dose ethanol (4 g/kg). All mice were housed under specific pathogen-free conditions with controlled temperature and 12 h light/dark circle. For the collection of tissue samples, mice were anesthetized with inhalational isoflurane. All animal studies were conducted by following the protocol approved by the North Carolina Research Campus Institutional Animal Care and Use Committee.

### Isolation of Primary Hepatocyte and Liver Immune Cells

Primary hepatocyte and liver immune cells were isolated, as previously described (Hao et al., 2020). Briefly, the livers were perfusion with cold PBS via the portal vein and followed by digested for 15 min at 37°C in digestion buffer. After digestion, dissociated cells were collected and filtered through the 100 μm cell strainer (BD Biosciences, San Jose, CA, United States) followed by centrifuge (50 g for 3 min at 4°C). Supernatant were collected for measuring the proportion of neutrophils. Pellets were suspended with 18 ml 40% ice-cold percoll (GE Healthcare, Bensalem, PA, United States) and centrifuged at 180 g for 5 min at 4°C. After this step, viable hepatocyte will be at the bottom of the tubes.

### Flow Cytometry

Cells (2 × 10^7^) were counted and resuspended in staining buffer containing murine Fc-block (CD16/32) antibody (BioLegend, Dedham, MA, United States) and incubated for 5 min on ice. For the detection of neutrophils cells in the liver, antibodies include CD45, CD11b, and Ly6g (BioLegend) were added to cells in flow cytometry staining buffer. After 25 min incubation in the dark on ice, 7-AAD (5 μl/per test; BioLegend) antibody were added and incubated for 5 min at room temperature to exclude dead cells. For total ROS generation measurement, 10 μM cell-permeant 2′,7′-dichlorodihydrofluorescein diacetate, H2DCFDA (Thermo Fisher Scientific, Waltham, MA, United States) were added to the medium. After 20 min incubation, cells were collected, and fluorescence intensity was measured. To detect mitochondrial ROS production, 1 μM MitoSOX Red Mitochondrial Superoxide Indicator (Thermo Fisher Scientific) was added to the medium for 15 min at 37°C. Teramethylrhodamine, Ethdl Ester, Perchlorate (TMRE) probe (Thermo Fisher Scientific) was used to determine the mitochondrial membrane potential detection according to the manufacturer’s instructions. Hepatocyte apoptosis was analyzed with an Annexin V probe (Biolegend) according to the manufacturer’s protocol. Briefly, hepatocytes were collected, washed with ice-cold PBS, and resuspended in 100 μl binding buffer staining containing 5 μl Annexin V probe for 15 min at room temperature in the dark. Following incubation, samples were analyzed using a BD FACSMelody flow cytometer (BD Bioscience). Data were analyzed with FlowJo software (TreeStar, Ashland, OR, United States).

### Cell Culture Study

AML12 cells were cultured in Dulbecco’s modified Eagle’s medium (Gibco, Waltham, MA, United States) supplementated with 10% FBS and 100 U/ml penicillin/streptomycin in cell culture flasks with solid caps (Thermo Scientific, Rockford, IL, United States). Overnight, cells were reached 80% confluence and treated with alcohol toxicants 100 μmol/L acetaldehyde (Sigma-Aldrich) for 24 h with or without 500 nM CpG ODN pretreatment for 6 h. For CRISPR transfection, 1.5 × 10^5^–2.0 × 10^5^ cells were seeded in a six wells culture plate with antibiotic-free standard growth medium, 24 h prior to transfection and cells were allowed to grow to a 40–60% confluency. CRISPR/Cas9 knockdown or control plasmids (Santa Cruz) were transfected into AML12 cells, respectively. Stable activated clone cells were selected. After selection, target gene knockdown cells were confirmed by RT-qPCR and Western blot analysis.

### Biochemical Analysis

Hepatic NAD^+^ contents were determined by a commercial kit (Biovision CA, United States) according to the manufacturer’s instructions as previously described (Song et al., 2020). The levels of triglycerides and free fatty acids in the liver were measured with Triglyceride Assay Kit and Free Fatty Acid Assay Kit (Biovision CA, United States), respectively, according to manufacturer’s instructions. Hepatic GSH levels were measured by a commercial kit (Biovision CA, United States) according to the manufacturer’s instructions. Serum ALT and AST activity was calorimetrically measured using Infinity kits (Thermo Scientific, MA, United States) according to the manufacturer’s instructions.

### Quantitative Real-Time PCR

Total RNA was isolated from the liver or hepatocytes were reverse-transcribed with TaqMan Reverse Transcription Reagents (Applied Biosystems, Foster City, CA, United States). The gene expression of related mRNAs was measured in triplicate by the comparative cycle threshold method using a 7500 real-time PCR system (Applied Biosystems, Foster City, CA, United States). The primer sequences (Integrated DNA Technologies, IL, United States) were shown in Supplementary Table S1. The data were normalized to 18s rRNA mRNA levels and presented as fold changes, setting the value of controls as 1.

### Western Blot

Protein lysates were extracted using lysis buffer supplemented with the protease inhibitor and phosphatase inhibitor (Sigma-Aldrich). Aliquots containing 50 μg of proteins were loaded onto an 8–12% SDS-PAGE, trans-blotted onto a PVDF membrane, blocked with 4% skimmed milk in Tris-buffered saline solution with 0.1% Tween-20 for 20 min at room temperature, and incubated with primary antibodies (Supplementary Table S2). Membranes were washed and incubated with secondary antibodies (Supplementary Table S2). Bound complexes were detected through enhanced chemiluminescence (GE Healthcare, Piscataway, NJ, United States). Bands intensity was quantified by ImageJ (NIH), and the ratio to β-actin was calculated and given as fold changes, setting the values of control groups at 1.

### Histological Analysis

#### Histopathology

Hematoxylin and eosin (H&E) staining was conducted with formalin-fixed liver tissue paraffin sections for observation of pathological changes.

#### Immunohistochemistry

Liver tissue paraffin sections were incubated with 3% hydrogen peroxide for 10 min to inactivate endogenous peroxidases and with normal serum for 20 min. Then the tissue sections were incubated with primary antibodies at 4°C overnight, followed by incubation with the corresponding secondary antibody (Agilant, Santa Clara, CA, United States) at room temperature for 30 min. Visualization was conducted with hydrogen peroxide and diaminobenzidine (DAB).

### Statistical Analysis

The analyses were performed using SPSS 19.0 software (SPSS, IL, United States). Data are expressed as the mean ± standard deviation (SD). Results were analyzed using the Student’s t-test or one-way analysis of variance (ANOVA) with Tukey’s post hoc test, where it was appropriate. In all tests, *p* values less than 0.05 were considered statistically significant.

## Results

### *TLR9* KO Mice Are More Susceptible to Chronic Alcohol Feeding-Induced Liver Injury and Steatosis

**FIGURE 1:**
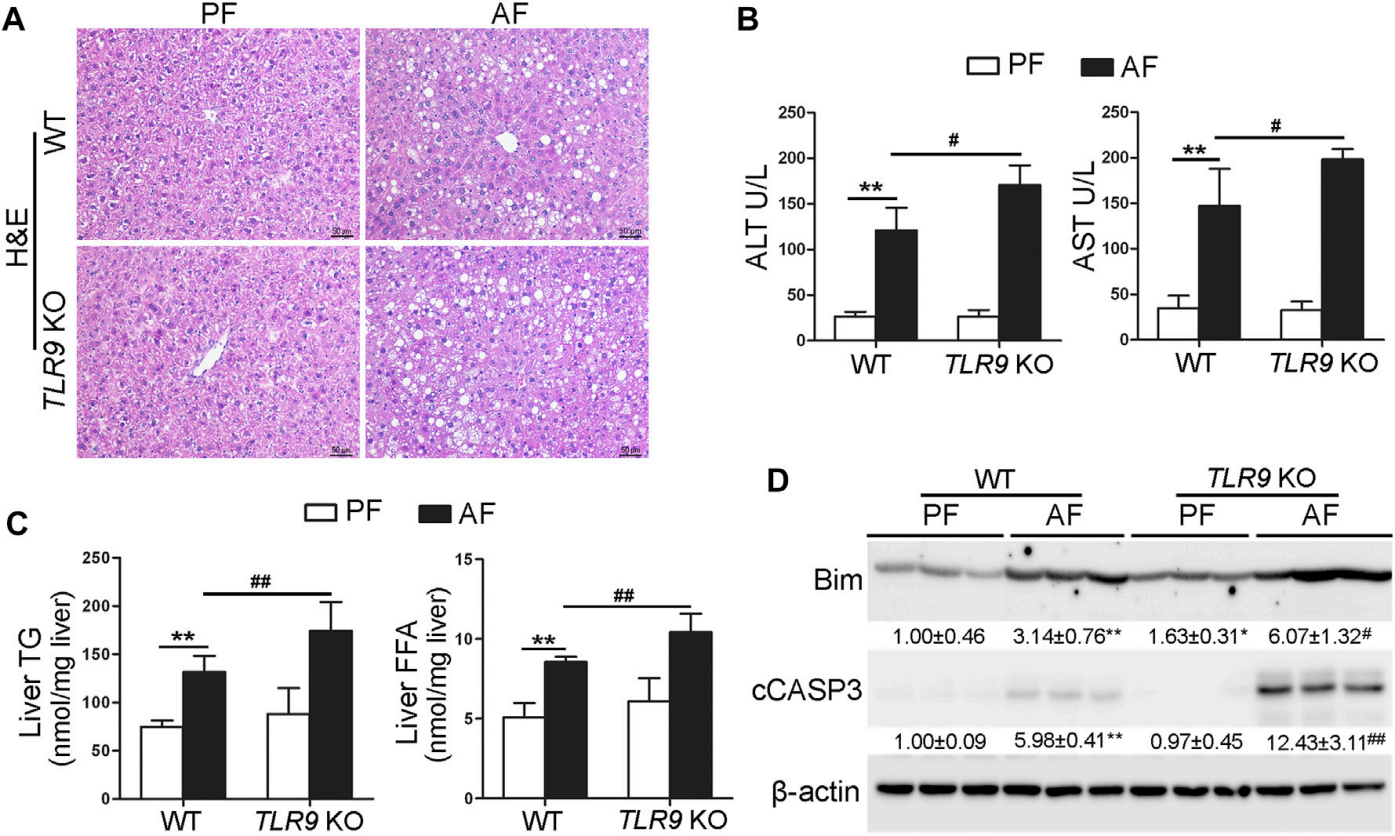
Chronic alcohol feeding-induced liver injury was exacerbated by *TLR9* KO. *TLR9* KO and wild-type male mice were pair-fed or alcohol-fed for 8 weeks plus a single binge of ethanol (4 g/kg) before 4 h of tissue collection. **(A)** Liver histopathological changes shown by H&E staining. Scale bars: 50 μm. **(B)** Serum ALT and AST activities. **(C)** Liver TG and FFA levels. **(D)** Western bolt analysis of hepatic Bim and cleaved caspase-3. Protein bands intensity was quantified by ImageJ (NIH). Data are presented as means ± SD. **p < 0.01 vs. WT/PF mice; ^#^ *p* < 0.05, ^##^ *p* < 0.01 vs. WT/AF mice. PF, pair-fed; AF, alcohol-fed.

TLR9 whole-body knockout (*TLR9* KO) mice were fed with alcohol for 8 weeks plus one binge to determine the role of TLR9 signaling in the pathogenesis of ALD. As shown in Figure 1A, liver histopathological changes induced by alcohol exposure, including lipid droplet accumulation, were exacerbated in *TLR9* KO mice. Alcohol-increased serum levels of alanine aminotransferase (ALT) and aspartate aminotransferase (AST) were also enhanced in *TLR9* KO mice (Figure 1B). Moreover, alcohol-increased hepatic triglyceride (TG) and free fatty acid (FFA) contents were exacerbated by *TLR9* KO (Figure 1C). Furthermore, alcohol-increased hepatic protein levels of Bcl-2-like protein 11 (Bim) and cleaved caspase-3 (cCASP3) were all exacerbated by *TLR9* KO (Figure 1D).

### Alcohol-Induced Neutrophils Infiltration in the Liver Was Ameliorated by *TLR9* KO

**FIGURE 2:**
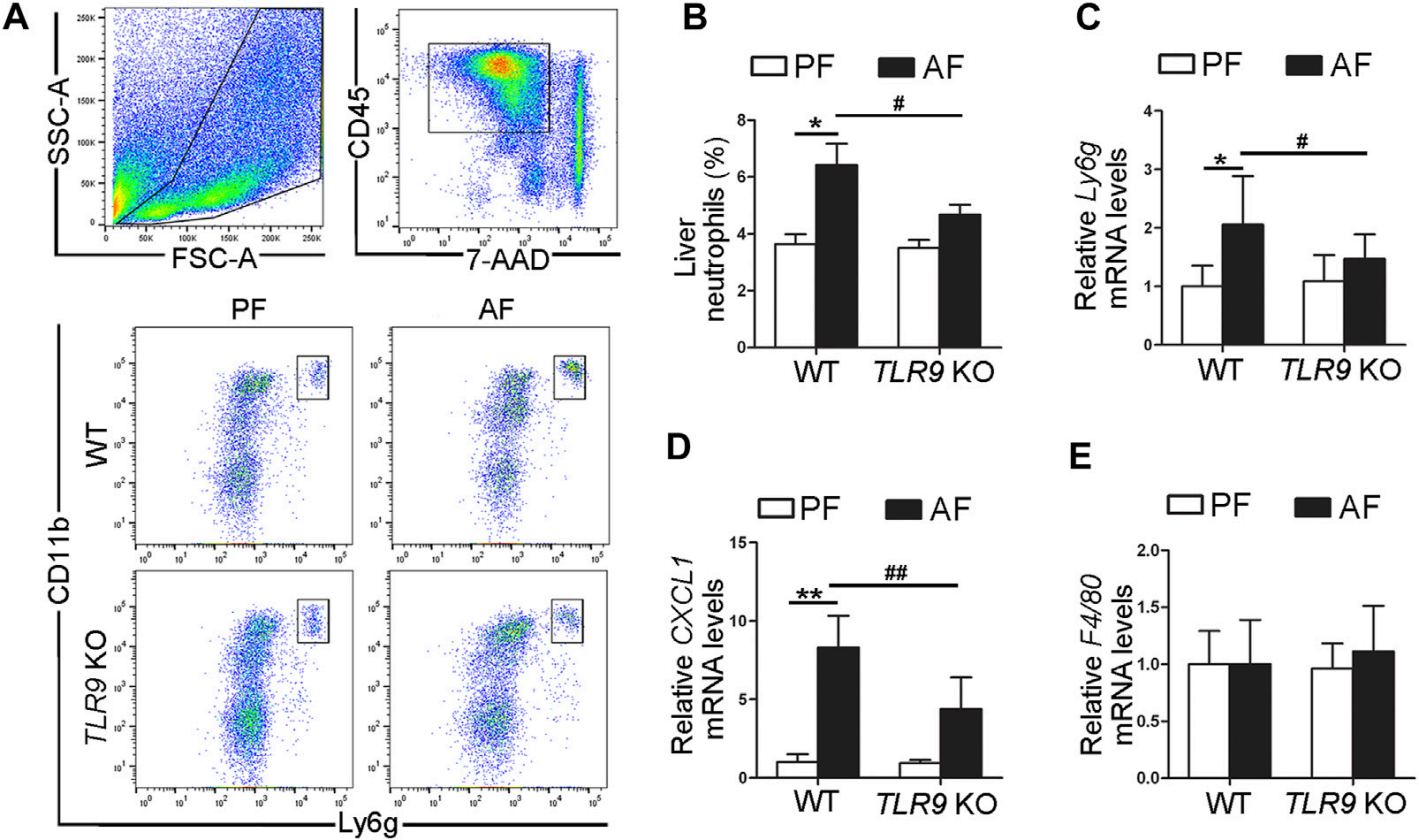
***TLR9*** deletion ameliorates alcohol-induced proinflammatory response in the liver. *TLR9* KO and wild-type male mice were pair-fed or alcohol-fed for 8 weeks plus a single binge of ethanol (4 g/kg) before 4 h of tissue collection. **(A)** Gating strategy of neutrophils infiltration in the liver. **(B)** Bar graph of liver neutrophils. **(C)** Bar graph of hepatic Ly6g mRNA levels. **(D)** Bar graph of hepatic CXCL1 mRNA levels. **(E)** Bar graph of hepatic F4/80 mRNA levels. Data are presented as means ± SD. *p < 0.05, **p < 0.01 vs. WT/PF mice; ^#^ *p* < 0.05, ^##^ *p* < 0.01 vs. WT/AF mice. PF, pair-fed; AF, alcohol-fed.

In contrast with the effects of TLR9 in alcohol-induced liver injury, alcohol-induced neutrophils (singlet/7-AAD^−^/CD45^+^/CD11b^+^/Ly6g^+^) infiltration in the liver were ameliorated by *TLR9* KO (Figures 2A,B). Compared with WT/PF mice, the mRNA levels of hepatic *Ly6g*, a neutrophils marker, were significantly increased in the livers of WT/AF mice; whereas this effect was ameliorated by *TLR9* deletion (Figure 2C). Consistently, alcohol-increased hepatic chemokine (C-X-C motif) ligand 1 (*CXCL1*) mRNA levels were also reversed by *TLR9* KO (Figure 2D). However, the mRNA levels of hepatic F4/80 were comparable between WT/AF mice and *TLR9* KO/AF mice (Figure 2E).

### Alcohol-Induced Hepatic Oxidative Stress and Mitochondrial Dysfunction Were Exacerbated in *TLR9* KO Mice

**FIGURE 3:**
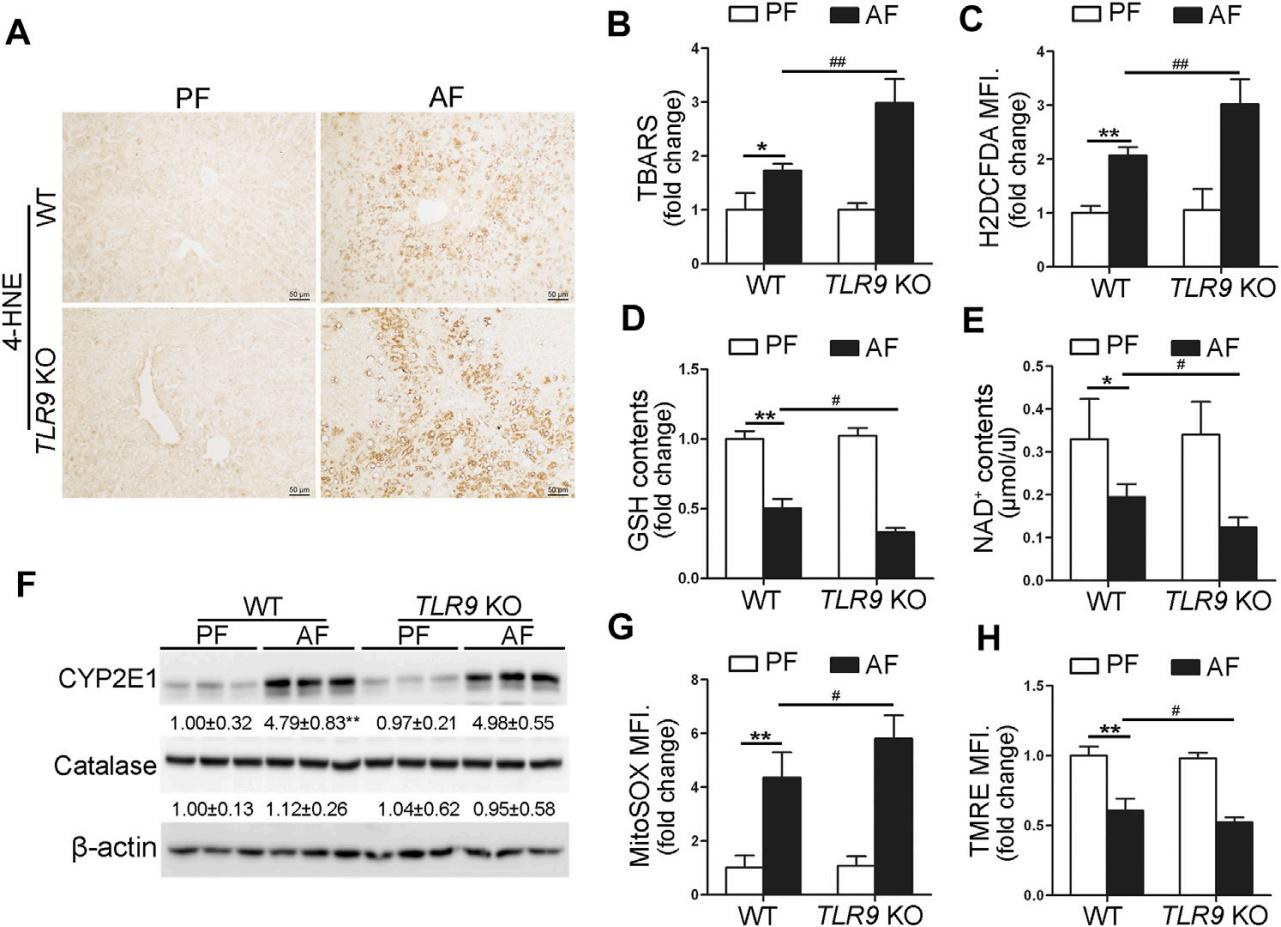
Alcohol-induced oxidative stress and mitochondrial dysfunction were exacerbated in *TLR9* KO mice. *TLR9* KO and wild-type male mice were pair-fed or alcohol-fed for 8 weeks plus a single binge of ethanol (4 g/kg) before 4 h of tissue collection. **(A)** Immunohistochemistry analysis of hepatic 4-HNE expression. Scale bars: 50 μm. **(B)** Hepatic TBARS levels. **(C)** FACS analysis of hepatic total ROS using H2DCFDA dye. Data are the summary of the mean fluorescence intensity (MFI). **(D)** Hepatic GSH levels. **(E)** Hepatic NAD^+^ levels. **(F)** Western bolt analysis of hepatic CYP2E1 and catalase. **(G)** FACS analysis of hepatic mitochondrial ROS using MitoSOX dye (*n* = 5). **(H)** FACS analysis of hepatic mitochondrial membrane potential using TMRE dye (n=5). Data are the summary of the mean fluorescence intensity (MFI). Data are presented as means ± SD. *p < 0.05, **p < 0.01 vs. WT/PF mice; ^#^ *p* < 0.05, ^##^ *p* < 0.01 vs. WT/AF mice. PF, pair-fed; AF, alcohol-fed.

We further determine if TLR9 signaling can affect alcohol-induced hepatic oxidative stress. As shown in Figure 3A, alcohol-increased 4-hydroxynonenal (4-HNE) protein adducts formation in the liver was exacerbated by *TLR9* KO. Consistently, alcohol-increased hepatic thiobarbituric acid reactive substances (TBARS) levels were also exacerbated in *TLR9* KO mice (Figure 3B). Hepatocytes isolated from WT/AF mice exhibited significantly higher total reactive oxygen species (ROS) levels than those from WT/PF mice, whereas this effect was exacerbated in *TLR9* KO mice (Figure 3C). Furthermore, alcohol-decreased hepatic glutathione (GSH) levels were enhanced in *TLR9* KO mice (Figure 3D). Alcohol-decreased hepatic nicotinamide adenine dinucleotide (NAD^+^) levels were also exacerbated by *TLR9* KO (Figure 3E). Hepatic Cytochrome P450 2E1 (CYP2E1) and catalase metabolizes ethanol leading to production of reactive oxygen species (ROS) and acetaldehyde, which are known to cause liver damage. However, the protein levels of hepatic CYP2E1 and catalase were comparable between WT/AF mice and *TLR9* KO/AF mice (Figure 3F). Intriguingly, alcohol-mediated mitochondrial ROS overgeneration was significantly enhanced in *TLR9* KO mice (Figure 3G). Moreover, alcohol-induced mitochondrial membrane potential disruption was also exacerbated in *TLR9* KO mice (Figure 3H).

### Activates Toll-Like Receptor 9 Ameliorates Acetaldehyde-Induced Oxidative Stress and Cell Injury in Hepatocyte

**FIGURE 4:**
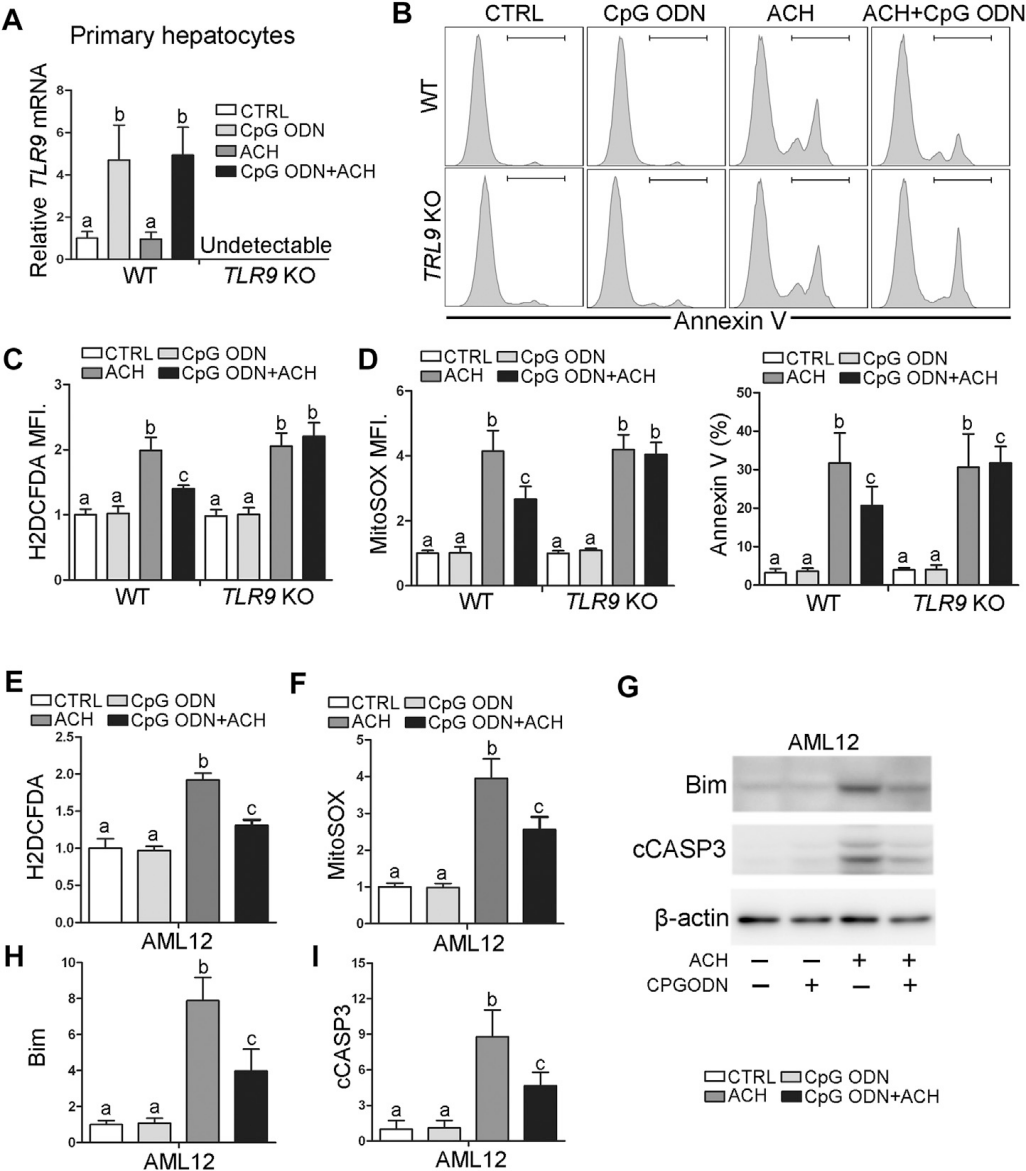
TLR9 activation ameliorates acetaldehyde-induced oxidative stress, mitochondrial dysfunction, and apoptosis in hepatocytes. **(A–D)** Primary hepatocytes isolated from wild-type and TLR9 KO mice were treated with CpG ODN and acetaldehyde (ACH). **(A)** Relative *TLR9* mRNA levels in primary hepatocytes. **(B)** FACS analysis of apoptotic cell death represented by Annexin V positive cells. **(C)** FACS analysis of hepatic total ROS using H2DCFDA dye. Data are the summary of the mean fluorescence intensity (MFI). **(D)** FACS analysis of hepatic mitochondrial ROS using MitoSOX dye. Data are the summary of the mean fluorescence intensity (MFI). **(E–I)** AML12 cells were treated with CpG ODN and ACH. **(E)** FACS analysis of hepatic total ROS using H2DCFDA dye. Data are the summary of the mean fluorescence intensity (MFI). **(F)** FACS analysis of hepatic mitochondrial ROS using MitoSOX dye. Data are the summary of the mean fluorescence intensity (MFI). **(G)** Western blotting of Bim and cCASP3. **(H–I)** Bar graphs of Bim and cCASP3. Protein bands intensity was quantified by ImageJ (NIH). Data are presented as means ± SD. Statistical comparisons were made using one-way ANOVA with Tukey’s post hoc test. Bars with different characters differ significantly (*p* < 0.05).

To evaluate whether TLR9 activation can protect acetaldehyde-induced oxidative stress and hepatocellular injury, primary hepatocytes were isolated from WT and *TLR9* KO mice, respectively, and treated with CpG oligodeoxynucleotides (CpG ODN) and acetaldehyde (ACH). As shown in Figure 4A, CpG ODN but not ACH significantly activates TLR9 signaling in primary hepatocytes isolated from WT mice, whereas this effect was abolished in cells from *TLR9* KO mice. Further flow cytometry analysis showed that pretreat with CpG ODN significantly ameliorated ACH-induced cell death, whereas this effect was abolished in *TLR9* KO mice (Figure 4B). Consistently, CpG ODN pretreatment also protects ACH-increased total ROS (Figure 4C) and mitochondria-derived ROS (Figure 4D) levels in primary hepatocytes isolated from WT mice but not *TLR9* KO mice. AML12 cells were used to further confirm the protective effect of CpG ODN on ACH-induced hepatocellular injury. Same as primary hepatocyte, ACH-induced oxidative stress, mitochondrial ROS overgeneration, and cell death were all ameliorated by CpG ODN pretreatment in AML12 cells (Figures 4E–H).

### CHOP Was Involved in the Protective Role of Toll-Like Receptor 9 Signaling in Acetaldehyde-Indued Cell Injury and Oxidative Stress

**FIGURE 5:**
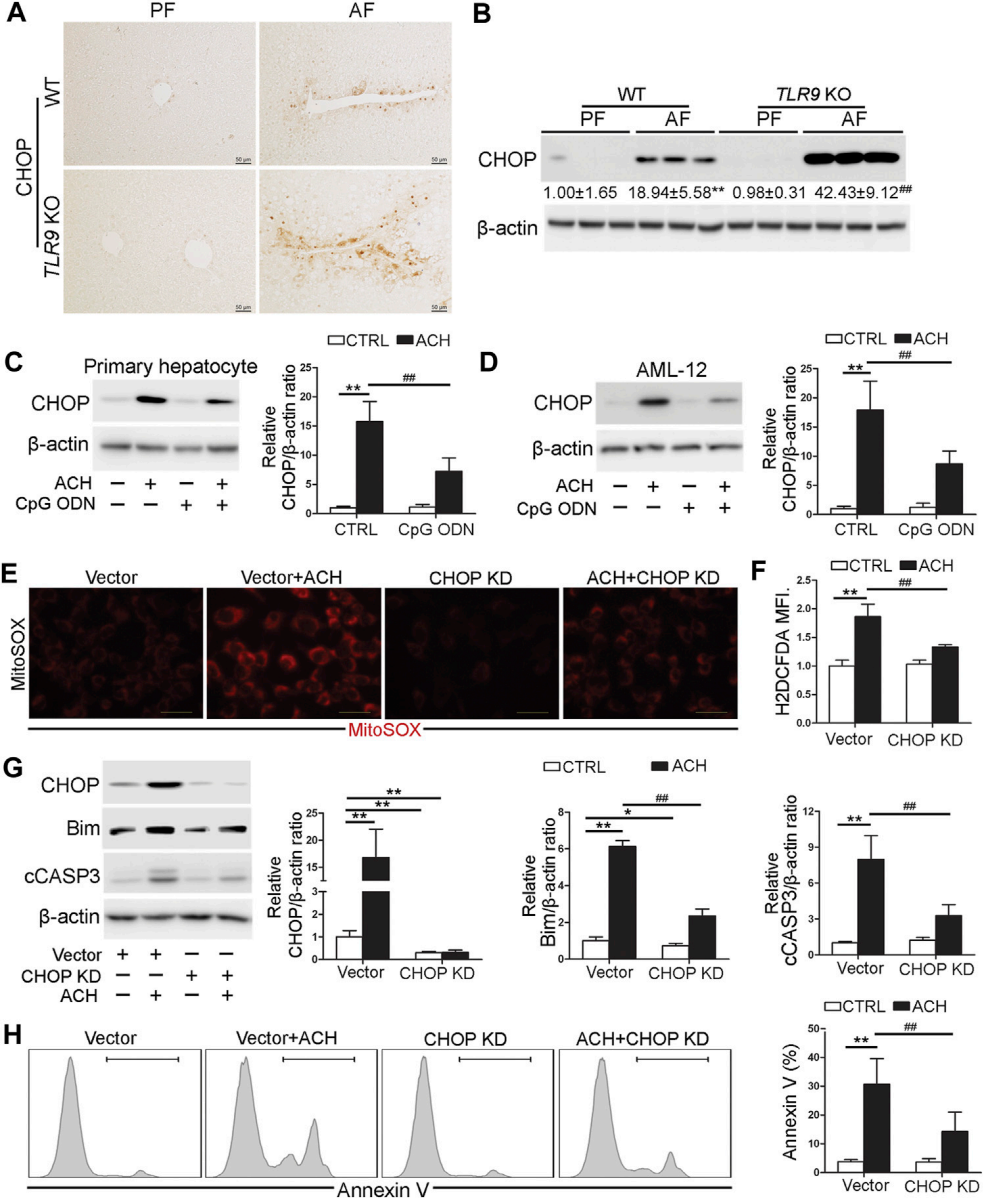
CHOP was involved in the protective role of TLR9 signaling in acetaldehyde-indued cell injury and oxidative stress. **(A,B)** *TLR9* KO and wild-type male mice were pair-fed or alcohol-fed for 8 weeks plus a single binge of ethanol (4 g/kg) before 4 h of tissue collection. **(A)** Immunohistochemistry analysis of hepatic CHOP expression. Scale bars: 50 μm. **(B)** Western boltting of hepatic CHOP. **(C)** CHOP protein levels in primary hepatocytes. **(D)** CHOP protein levels in AML12 cells. **(E)** Immunofluorescent staining of mitochondrial ROS using MitoSOX probe. **(F)** FACS analysis of hepatic total ROS using H2DCFDA dye. Data are the summary of the mean fluorescence intensity (MFI). **(G)** Western bolt analysis of CHOP, Bim, and cCASP3 levels in AML12 cells. **(H)** FACS analysis of apoptotic cell death represented by Annexin V positive cells. **(A,B)** **p < 0.01 vs. WT/PF mice; ^##^ *p* < 0.01 vs. WT/AF mice. PF, pair-fed; AF, alcohol-fed. **(C–H)** *p < 0.05, **p < 0.01 vs. control; ^##^ *p* < 0.01 vs. ACH treated cells.

Both IHC and western blotting analysis indicated that alcohol-induced hepatic C/EBP homologous protein (CHOP) activation was strongly exacerbated in *TLR9* KO mice (Figures 5A,B). We further found that both primary hepatocyte and AML12 cells pretreat with CpG ODN ameliorated ACH-induced CHOP activation (Figures 5C,D). To confirm whether CHOP activation plays a detrimental role in ACH-induced oxidative stress and cell death, we generated *CHOP* knockdown (KD) AML12 cell lines by using the CRISPR/Cas9 system. As shown in Figures 5E,F, ACH-increased mitochondrial ROS and total ROS were significantly ameliorated by *CHOP* KD in AML-12 cells (Figures 5E,F). Furthermore, ACH-increased protein levels of Bim and cCASP3 were also significantly ameliorated by CHOP KD in AML-12 cells (Figure 5G). Consistently, ACH-increased frequency of Annexin V positive cells were also significantly decreased by CHOP KD (Figure 5H).

### Toll-Like Receptor 9 Control CHOP Activation *via* Regulating ATF6 in Hepatocyte

**FIGURE 6:**
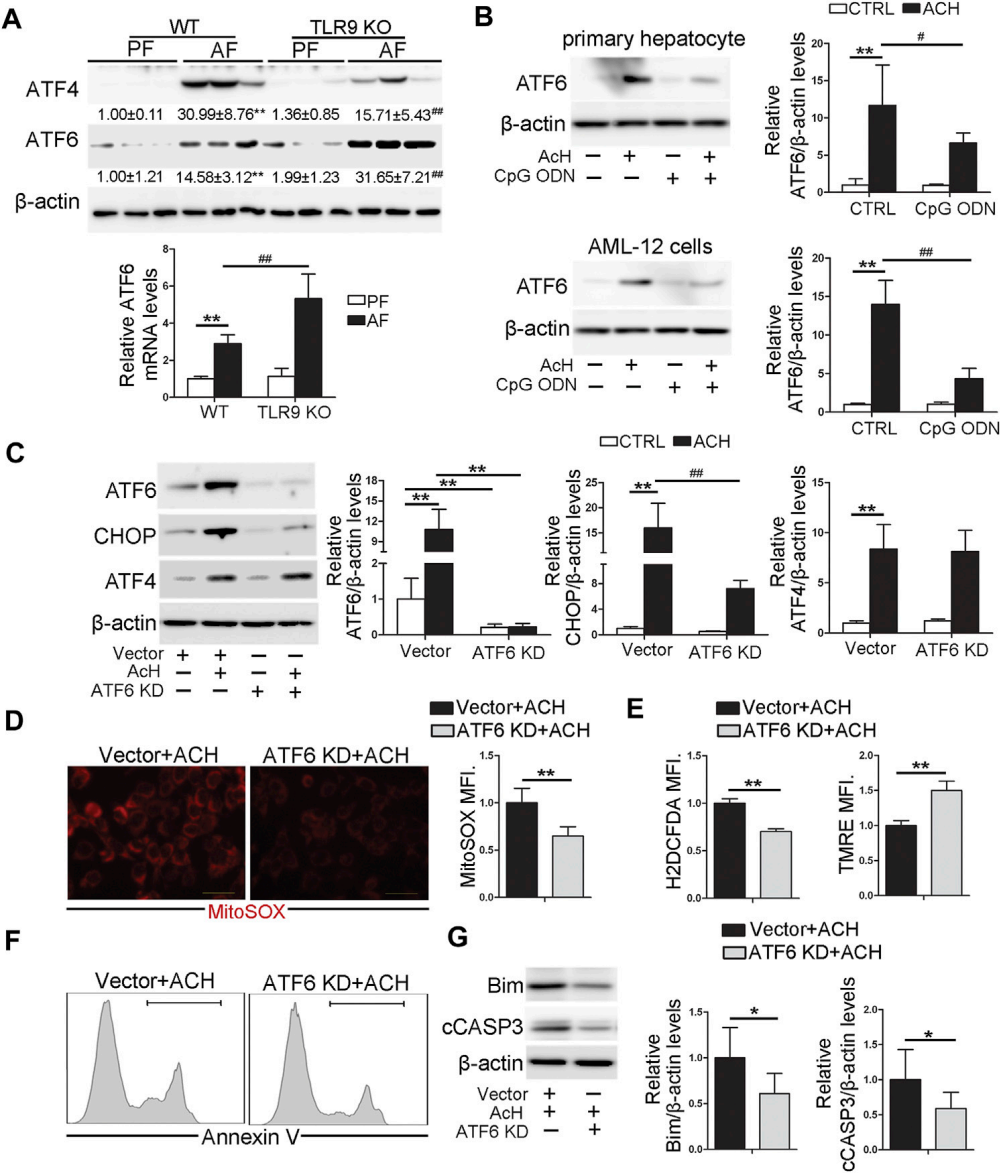
TLR9 control CHOP activation via regulating ATF6 in hepatocyte. **(A)** *TLR9* KO and wild-type male mice were pair-fed or alcohol-fed for 8 weeks plus a single binge of ethanol (4 g/kg) before 4 h of tissue collection. Hepatic ATF4 and ATF6 protein levels and relative ATF6 mRNA levels in mice. **(B)** ATF6 protein levels in primary hepatocytes and AML12 cells. **(C)** Protein levels of ATF6, CHOP, and ATF4 in ATF6 knockout AML12 cells. **(D)** Immunofluorescent staining and flow cytometry analysis of mitochondrial ROS using MitoSOX probe. **(E)** FACS analysis of hepatic total ROS and mitochondrial membrane potential. **(F)** FACS analysis of apoptotic cell death represented by Annexin V positive cells. **(G)** Western blotting of Bim and cCASP3. **(A)** **p < 0.01 vs. WT/PF mice; ^##^ *p* < 0.01 vs. WT/AF mice. PF, pair-fed; AF, alcohol-fed. **(B,C)** **p < 0.01 vs. Control cells; ^#^ *p* < 0.05, ^##^ *p* < 0.01 vs. ACH treated cells. **(D–G)** * < *p*0.05, **p < 0.01 vs. ACH treated cells.

Previous studies demonstrated that both ATF4 and ATF6 can directly control the transcription activity of CHOP (Fusakio et al., 2016; Yang et al., 2020). In this study, we found that the protein levels of hepatic ATF4 and ATF6 were both significantly increased by chronic alcohol feeding (Figure 6A). However, compared with WT/AF mice, the protein levels of hepatic ATF4 were significantly decreased in *TLR9* KO/AF mice (Figure 6A). Interestingly, share the same trend as hepatic CHOP, alcohol-increased ATF6 protein levels were strongly enhanced by *TLR9* KO (Figure 6A), suggesting ATF6 but not ATF4 may be involved in CpG ODN-TLR9 signaling controlled CHOP expression. Indeed, ACH-induced ATF6 activation was significantly ameliorated by CpG ODN pretreatment in both primary hepatocytes and AML-12 cells (Figure 6B). Moreover, ATF6 KD significantly reversed ACH-induced CHOP activation in AML-12 cells (Figure 6C). ACH-increased mitochondrial ROS and total ROS were ameliorated by ATF6 KD in AML-12 cells (Figures 6D,E). ACH-disrupted mitochondrial membrane potential was also meliorated by ATF6 KD (Figure 6E). Moreover, ACH-increased frequency of Annexin V positive cells were also ameliorated by ATF6 KD (Figure 6F). Compared with ACH-treated cells, ACH-treated ATF6 KD cells display significantly lower protein levels of Bim and cCASP3 in AML-12 (Figure 6G).

### Toll-Like Receptor 9 Control ATF6-CHOP Axis Expression *via* Regulating STAT3 Activation in Hepatocyte

**FIGURE 7:**
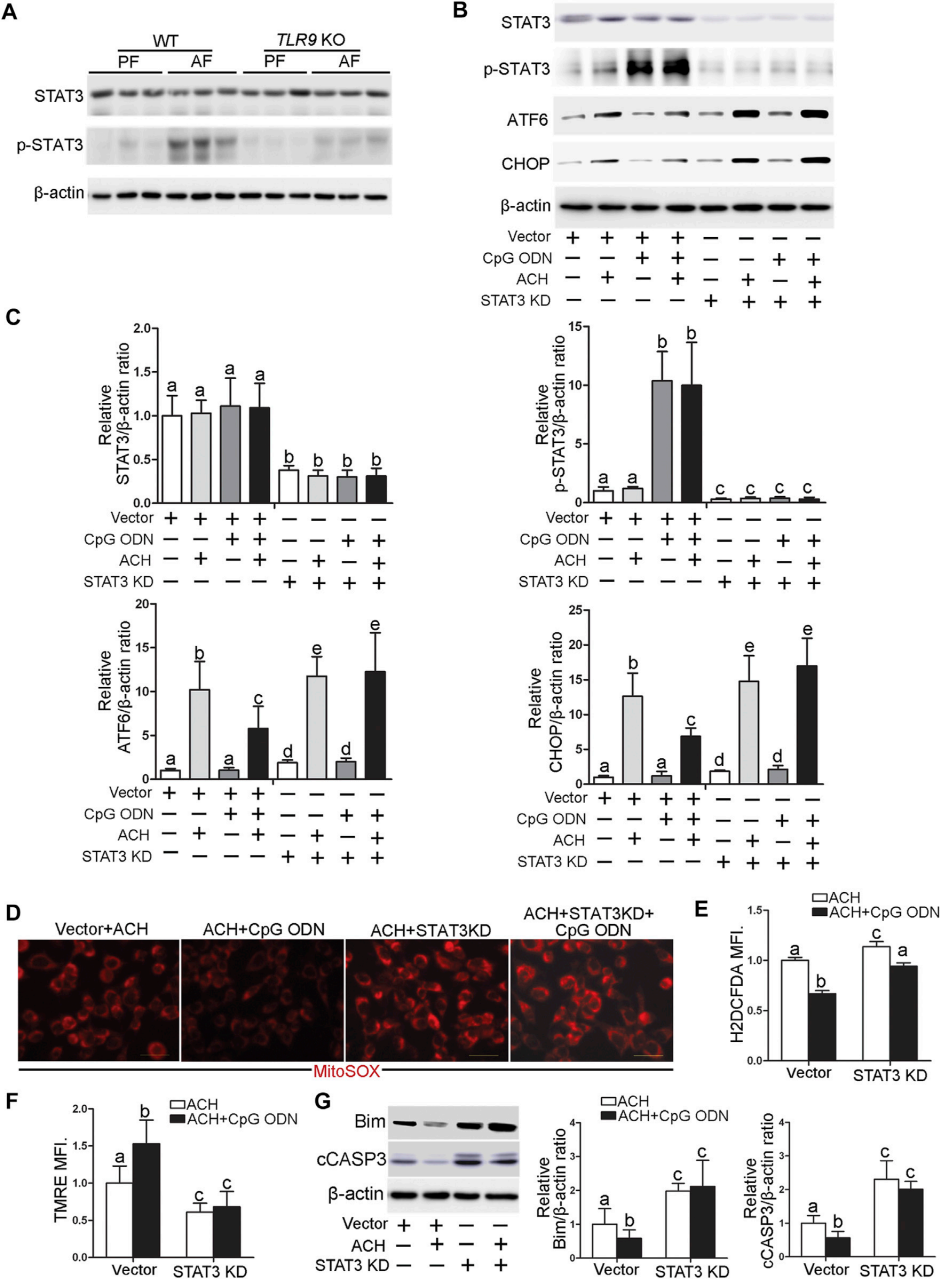
TLR9 control ATF6-CHOP axis expression via regulating STAT3 activation in hepatocyte. **(A)** *TLR9* KO and wild-type male mice were pair-fed or alcohol-fed for 8 weeks plus a single binge of ethanol (4 g/kg) before 4 h of tissue collection. Hepatic STAT3 and p-STAT3 protein levels in mice. **(B,C)** Protein levels of STAT3, p-STAT3, ATF6, and CHOP in STAT3 knockdown AML12 cells. **(D)** Immunofluorescent staining of mitochondrial ROS using MitoSOX probe. **(E)** FACS analysis of total ROS using H2DCFDA dye. **(F)** FACS analysis of mitochondrial membrane potential using TMRE dye. **(G)** Protein levels of Bim and cCASP3. Data are presented as means ± SD. Statistical comparisons were made using one-way ANOVA with Tukey’s post hoc test. Bars with different characters differ significantly (*p* < 0.05).

STAT3 activation has been reported to play an essential role in protecting against hepatocellular damage in multiple models. We found that alcohol-increased the hepatic phosphorylated STAT3 (p-STAT3) levels were significantly decreased in TLR9 KO mice (Figure 7A). Furthermore, CpG ODN-increased STAT3 activation was abolished by TLR9 KO (Figures 7B,C). To measure whether STAT3 activation was involved in the protective role of TLR9 activation in acetaldehyde-induced ATF6-CHOP signaling activation, STAT3 knockdown cells were generated and treated with acetaldehyde and CpG ODN. As shown in Figures 7B,C, *STAT3* knockdown sufficient lead to ATF6-CHOP signaling activation in hepatocyte. Moreover, the protective role of CpG ODN in acetaldehyde-induced ATF6-CHOP signaling activation was abolished in *STAT3* KD cells (Figures 7B,C). We next found that the protective role of CpG ODN on acetaldehyde-induced mitochondrial ROS overgeneration and mitochondrial membrane potential disruption was abolished by STAT3 KD (Figures 7D,F). Furthermore, the protective role of CpG ODN on ACH-induced total ROS overgeneration (Figure 7E) and cell death (Figure 7G) were also abolished by *STAT3* KD.

### STAT3 Positively Control *CXCL1* Expression in Hepatocyte

**FIGURE 8:**
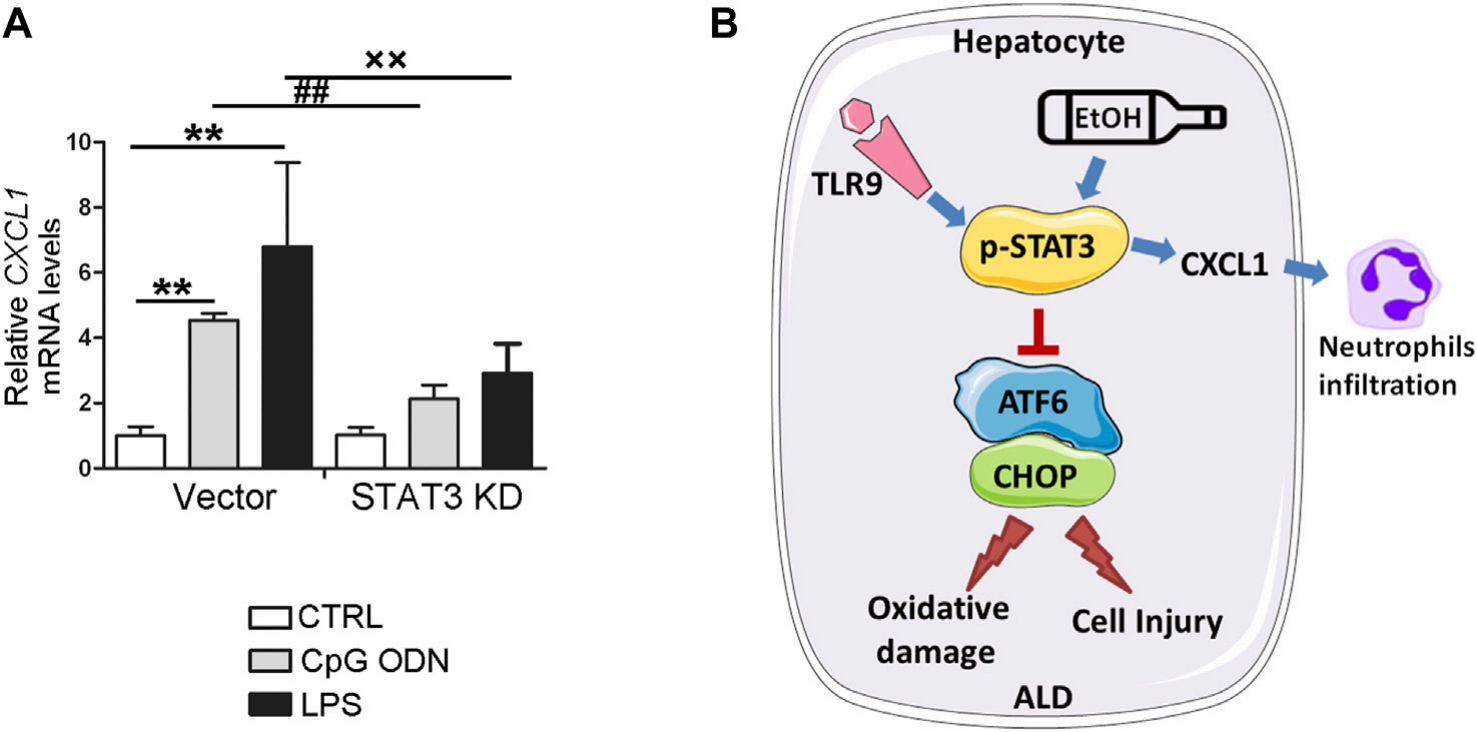
STAT3 positively control *CXCL1* expression in AML12 cells. **(A)** Relative *CXCL1* mRNA levels in *STAT3* knockdown AML12 cells. **p < 0.01 vs. Control cells; ^##^ *p* < 0.01 vs. CpG ODN treated cells; ××*p* < 0.01 vs. LPS treated cells. **(B)** Summarized Figure of this study.

STAT3 has been known as a key mediator for proinflammatory gene expression induced by pathogen-associated molecular patterns (PAMPs). To further explore whether STAT3 activation may be involved in the protective role of *TLR9* KO in *CXCL1* activation, *STAT3* KD cells were treated with CpG ODN and LPS. As shown in Figure 8A, both LPS and CpG ODN administrations were significantly increased the *CXCL1* mRNA levels in the hepatocyte, whereas these effects were reversed in *STAT3* KD hepatocyte, suggesting STAT3 was involved in the protective role of *TLR9* KO in alcohol-mediated hepatic *CXCL1* induction and neutrophils infiltration.

## Discussion

The present study demonstrated, for the first time, that whole-body *TLR9* deficiency exacerbates chronic alcohol-induced liver injury, steatosis, oxidative stress, mitochondrial dysfunction, and apoptosis. CpG OND, a TLR9 agonist, pretreatment protects acetaldehyde-induced mitochondrial dysfunction, oxidative stress, and apoptosis in both primary hepatocyte and AML12 cells. Investigations of the underlying mechanisms revealed that hepatic CHOP activation, resulting from ATF6 activation, is mechanistically involved in *TLR9* deficiency-mediated oxidative stress, mitochondrial dysfunction, and apoptosis in hepatocytes treated with acetaldehyde. Interestingly, we found that hepatic STAT3, which controls by CpG ODN-TLR9 signaling, plays a protective role in alcohol-induced ATF6-CHOP signaling activation, mitochondrial dysfunction, and apoptosis but exacerbates proinflammatory response in hepatocyte (Figure 8B).

TLR9 is a pattern recognition receptor that participates in host defense by recognizing pathogen-associated molecular patterns alongside inflammatory processes by recognizing damage-associated molecular patterns (Bauer et al., 2001). In this study, we identified that lack of TLR9 signaling exacerbated chronic alcohol exposure-induced liver injury. These results are indirectly in contrast with a previous report that *TLR9* KO mice protect alcoholic liver disease in mice (Roh et al., 2015). These controversies are most likely due to the difference in length (10 days in the previous report and 8 weeks in the current study) and method of alcohol exposure. Mechanically, we also found that *TLR9* KO mice exacerbated alcohol-induced oxidative stress and mitochondrial dysfunction. Furthermore, CpG ODN-mediated TLR9 activation in hepatocyte protects acetaldehyde-induced oxidative stress, mitochondrial dysfunction, and cell death. Consistent with our results, CpG ODN-mediated TLR9 activation protects tert-butyl hydroperoxide-induced mitochondrial dysfunction, oxidative stress, and cell injury in macrophages (Qu et al., 2020). Similarly, CpG ODN-mediated TLR9 signaling prevents shock-mediated death (Kim et al., 2008). *TLR9* deficiency also exhibited enhanced hepatic steatosis after high fat diet feeding in mice (Hong et al., 2015). However, one study reported that hepatocyte mitochondrial DNA drives steatohepatitis by activation of TLR9 (Garcia-Martinez et al., 2016). These results suggesting that the role of TLR9 signaling on cell injury might be dependent on the stress type and cell type.

Mitochondrial dysfunction is a hallmark of ALD. During the initiation and progression of ALD, hepatic mitochondrial dysfunction is intimately associated with liver damage in both human and mice (Mansouri et al., 2018). Our previous data demonstrated that mitochondrial ROS overgeneration plays a detrimental role in the development of alcohol-induced hepatic oxidative stress, steatosis, inflammation, and cell death (Lu et al., 2016; Hao et al., 2018). Previous studies, including ours, also indicated that ER stress is a pathological factor in alcohol-induced mitochondrial dysfunction (Hao et al., 2020). Notably, ER stress-induced CHOP activation under maternal diabetic conditions suppresses mitochondrial biogenesis and leads to accumulation of damaged mitochondria which causes mitochondrial dysfunction and the mitochondrial-dependent apoptosis in neuroepithelial cells (Chen et al., 2017). Furthermore, *CHOP* KO mice protects alcohol-induced cell death and liver injury (Ji et al., 2005), suggesting alcohol-induced CHOP activation plays a detrimental role in the pathogenesis of ALD. In this study, we found that CHOP activation is negatively regulated by TLR9 signaling and CHOP activation promotes alcohol-induced mitochondrial dysfunction in hepatocyte. Our results indicated that *CHOP* knockdown significantly ameliorated alcohol-induced mtROS overgeneration, mitochondrial membrane potential disruption, and cell death (Li et al., 2016b). Consistently, the previous study also demonstrated that CHOP activation serves as a negative regulator of mitochondrial biogenesis by directly suppressing peroxisome proliferator-activated receptor gamma coactivator 1-alpha (PGC-1α) transcription (Chen et al., 2017). Therefore, further studies are needed to explore whether PGC-1α reduction was involved in CHOP-mediated mitochondrial dysfunction in hepatocyte.

Previous studies have reported that hepatic ATF4, an upstream regulator of CHOP, is activated after chronic alcohol exposure and is involved in mitochondrial dysfunction (Li et al., 2016a). Unexpectedly, unlike the trend of hepatic CHOP, we found that TLR9 deficiency did not exacerbate alcohol-induced ATF4 activation in the mouse liver. Furthermore, by using ATF4 hepatocyte-specific knockout mice, we previously found that hepatic abrogation of ATF4 did not affect CHOP protein levels in the liver regardless of alcohol exposure (Hao et al., 2020). These results suggested that ATF4 is dispensable for CHOP induction in mice chronic fed with alcohol. Furthermore, lack of ATF4 in intestinal epithelial cells even leads to CHOP induction in the gut (Hu et al., 2019). Notably, a growing number of studies indicated that the ATF6 branch of the UPR is also a critical determinant of CHOP dynamics during ER stress (Hu et al., 2018). Indeed, our results showed that mice lack of TLR9 signaling in the liver significantly increased the protein levels of ATF4 in the mouse liver. By using the ATF6 deficiency cell lines we confirmed that ATF6 is a major regulator control CHOP activation during alcohol-mediated ER stress in hepatocyte. Similar to CHOP knockout in the hepatocyte, our results indicated that ATF6 deficiency cells protect alcohol-induced oxidative stress, mitochondrial dysfunction, and cell death. However, whether ATF6 activation also plays a detrimental role in alcohol-induced liver injury and mitochondrial dysfunction needs to be further explored in mice.

STAT3 is a master transcriptional factor involved in a broad spectrum of adaptive and innate immune functions (Fu, 2006; Ning et al., 2016). STAT3 is activated by a variety of factors, including cytokines, growth factors, hormones, and hepatitis viral proteins in the liver (Moh et al., 2007). A previous study demonstrated that STAT3 activation plays a protective role in hepatocellular damage in many models of liver injury (Wang et al., 2011; Li et al., 2013; Kwon et al., 2014). However, hepatic STAT3 also aggravate liver inflammation by promoting hepatocytes to secrete acute-phase proteins (Ahyi et al., 2013) and ablation of hepatic STAT3 reduced liver inflammation in mice (Ozturk Akcora et al., 2020). In this study, we found that STAT3 is an important regulator of *CXCL1* expression in CpG OND treated hepatocyte. Firstly, we found that *TLR9* KO mice protect alcohol-increased *CXCL1* mRNA levels and neutrophils infiltration in the liver. Secondly, we found that alcohol-mediated STAT3 activation was significantly reversed in *TLR9* KO mice. Finally, we found that cells lack of *STAT3* expression lower mRNA levels of *CXCL1* in both CpG ODN and LPS treated hepatocyte. Previous study demonstrated that hepatocyte-specific *STAT3* deletion exacerbates alcohol-induced steatosis and liver injury (Wang et al., 2011). In this study, our results showed that STAT3 activation also protects alcohol-induced oxidative stress, mitochondrial dysfunction and cell death in hepatocyte, suggesting STAT3 activation protects cell injury caused by ethanol administration. However, we could not rule out any other factors that may be involved in the protective role of TLR9 signaling in alcohol-induced cell injury and future studies are needed to test this point.

ALD ranks among the major causes of all liver diseases, affecting millions of patients worldwide. Unfortunately, no new drugs for ALD have been successfully developed over the past few decades. In this study, our data demonstrate that hepatic TLR9 signaling protects alcohol-induced oxidative stress by maintaining mitochondrial functions. Mechanistically, TLR9 signaling negatively regulates CHOP expression via repressing ATF6 activation in the hepatocytes. We showed that STAT3 activation is involved in the protective role of TLR9 activation in alcohol-induced oxidative stress, mitochondrial dysfunction, and apoptosis but promotes proinflammatory chemokine release in hepatocyte. In conclusion, our results reveals TLR9 have different roles in regulating alcohol-induced oxidative stress and inflammation in mouse liver. Future studies are required to explore the clinical potential of this approach.

## Data Availability

The raw data supporting the conclusion of this article will be made available by the authors, without undue reservation.
