# Supplementary material for: TLR9 Signaling Protects Alcohol-Induced Hepatic Oxidative Stress but Worsens Liver Inflammation in Mice
Source: Front Pharmacol. 2021 Jun 28;12:709002. doi: 10.3389/fphar.2021.709002 (PMC8273378; doi:10.3389/fphar.2021.709002)
Supplement: Supplementary file 1 [file DataSheet1.docx]

**TLR9 signaling protects alcohol-induced hepatic oxidative stress but worsens liver inflammation in mice.**

Liuyi Hao^1^, Wei Zhong^1,2^, Xinguo Sun^1^, Zhanxiang Zhou^1,2*^.

Affiliations: 1 Center for Translational Biomedical Research and 2 Department of Nutrition, the University of North Carolina at Greensboro, North Carolina Research Campus, Kannapolis, NC, USA.

* Corresponding author: Zhanxiang Zhou, Center for Translational Biomedical Research and Department of Nutrition, the University of North Carolina at Greensboro, North Carolina Research Campus, 600 Laureate Way, Suite 2203, Kannapolis, NC 28081. Phone: 704-250-5800 Fax: 704-250-5809 E-mail: z_zhou@uncg.edu

**Supplementary tables**

**Supplementary Table 1. Primers design for Real-Time PCR.**

| Name | | Forward | Reverse |
| --- | --- | --- | --- |
| CXCL1 | CATGGCTGGGATTCACCTCA | | GAGCTTCAGGGTCAAGGCAA |
| Ly6g | TGTGCAGAAAGAGCTCAGGG | | AACCAGGCTGAACAGAAGCA |
| F4/80 | TCTGGGGAGCTTACGATGGA | | GAATCCCGCAATGATGGCAC |
| TLR9 | GCGCCCAAACTCTCCCTTAT | | ATCTCGGTCCTCCAGACACA |
| ATF6 | GAACTTCGAGGCTGGGTTCA | | TCCAGGGGAGGCGTAATACA |

**Supplementary Table 2. Antibodies.**

| Antigen | Origin | Vendor | Catalog No. |
| --- | --- | --- | --- |
| Bim | Rabbit | Cell signaling technology | 2933S |
| Cleaved Caspase3 | Rabbit | Cell signaling technology | 9664S |
| CYP2E1 | Rabbit | Abcam | ab28146 |
| Catalase | Rabbit | Calbiochem | 219010 |
| CHOP | Rabbit | Cell signaling technology | 5554S |
| ATF4 | Rabbit | Cell signaling technology | 11815S |
| ATF6 | Rabbit | Cell signaling technology | 65880 |
| STAT3 | Mouse | Cell signaling technology | 9139S |
| P-STAT3 | Rabbit | Cell signaling technology | 9145S |

**Supplementary Figure 1**

**
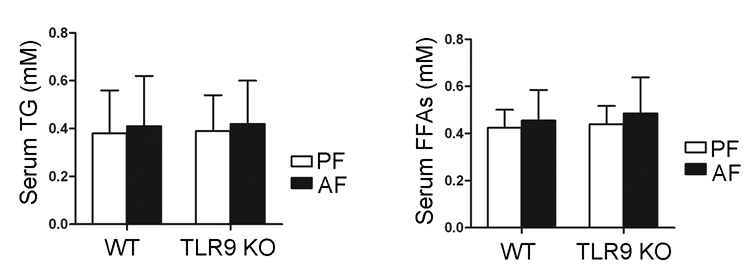
**

Supplementary Figure 1. The role of TLR9 in serum TG and FFAs levels. PF, pair-fed; AF, alcohol-fed.

**Supplementary Figure 2**


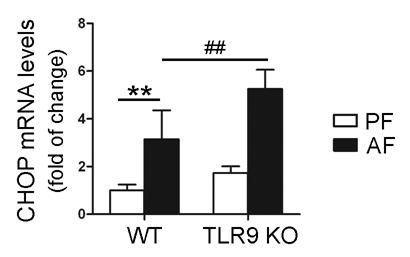


Supplementary Figure 2. The role of TLR9 in hepatic CHOP mRNA levels. Data are presented as means ± SD. **P<0.01 vs. WT/PF mice; ##P<0.01 vs. WT/AF mice. PF, pair-fed; AF, alcohol-fed.
